# Association between Serum Zinc and All-Cause Mortality in Patients Undergoing Maintenance Hemodialysis: The Osaka Dialysis Complication Study (ODCS)

**DOI:** 10.3390/nu16193270

**Published:** 2024-09-27

**Authors:** Shinya Nakatani, Tetsuo Shoji, Fumiyuki Morioka, Rino Nakaya, Mayuko Ueda, Hideki Uedono, Akihiro Tsuda, Tomoaki Morioka, Hisako Fujii, Hisako Yoshida, Katsuhito Mori, Masanori Emoto

**Affiliations:** 1 Department of Metabolism, Endocrinology and Molecular Medicine, Osaka Metropolitan University Graduate School of Medicine, Osaka 545-8585, Japan; fumimorioka0509@gmail.com (F.M.); 828rino828@gmail.com (R.N.); uedono1217@omu.ac.jp (H.U.); naranotsudadesu@omu.ac.jp (A.T.); moriokatmed@omu.ac.jp (T.M.); emoto-m@omu.ac.jp (M.E.); 2 Department of Vascular Medicine, Osaka Metropolitan University Graduate School of Medicine, Osaka 545-8585, Japan; tetsuoshoji@omu.ac.jp; 3 Vascular Science Center for Translational Research, Osaka Metropolitan University Graduate School of Medicine, Osaka 545-8585, Japan; 4 Department of Medical Statistics, Osaka Metropolitan University Graduate School of Medicine, Osaka 545-8585, Japan; sn23119e@st.omu.ac.jp (M.U.); hisako.yoshida@omu.ac.jp (H.Y.); 5 Department of Health and Medical Innovation, Osaka Metropolitan University Graduate School of Medicine, Osaka 545-8585, Japan; hfujii@omu.ac.jp; 6 Department of Nephrology, Osaka Metropolitan University Graduate School of Medicine, Osaka 545-8585, Japan; ktmori@omu.ac.jp

**Keywords:** zinc, hemodialysis, mortality

## Abstract

Background/Objectives: Zinc is an essential microelement, and its deficiency is common in patients undergoing hemodialysis. However, the association between serum zinc and mortality in these patients remains unclear. The aim of this study was to explore the possible association between serum zinc levels and all-cause mortality in prevalent patients with kidney failure on maintenance hemodialysis. Methods: This was a prospective cohort study of maintenance hemodialysis patients followed up for 5 years. The key exposure was serum zinc level measured at baseline, and the outcome was all-cause mortality. Their association was analyzed using Cox proportional hazard models. Results: Among 1662 eligible patients selected for this analysis, 468 (28%) died. Lower serum zinc levels were associated with a higher risk for mortality, independent of the major demographic factors and factors including mineral and bone disorder and renal anemia. However, this association was no longer significant when adjusted for serum albumin. Because there was a close correlation between serum zinc and albumin levels, we performed further analyses in which participants were categorized into four groups by median serum zinc (68 µg/dL) and albumin (3.7 g/dL) levels. In the lower serum albumin groups, risk of death was significantly higher in those with lower zinc than those with higher zinc levels, whereas such a difference was not significant in the high serum albumin groups. Conclusions: In patients undergoing maintenance hemodialysis with lower serum albumin levels, a lower serum zinc level was associated with a higher risk of mortality.

## 1. Introduction

Zinc, one of the micronutrients, is an essential trace element and the second most abundant divalent cation in the body. In human, 60% of zinc is stored in skeletal muscle and 20% in bones, while the circulating zinc accounts for only 0.1% of total body zinc [1]. In the circulation, 80% of zinc is distributed in erythrocytes and 20% in plasma, which is predominantly bound to several proteins such as albumin, α-macroglobulin, and transferrin [2]. Reduced levels of zinc in plasma or serum of patients with chronic kidney disease (CKD) including those undergoing hemodialysis have been demonstrated [3,4]. Zinc deficiency in those patients is due to several factors including decreased food intake, interstitial malabsorption, increased exclusion into urine and feces, and removal by hemodialysis [5,6].

Zinc plays key roles for various biological processes as a cofactor with ≥300 enzymes including alcohol dehydrogenase, alkaline phosphatase (ALP), angiotensin converting enzyme, carbonic anhydrase, collagenase, lactate dehydrogenase (LDH), DNA polymerase, and RNA polymerase [7]. Thus, zinc is involved in the regulation of alcohol metabolism, bone metabolism, glucose metabolism, and blood pressure control [7,8,9,10]. Zinc also plays important roles in the regulation of immune functions of many cells (T, B and natural killer) [11]. Zinc is essential in the active site of superoxide dismutase (SOD), an important antioxidant enzyme that catalyzes the dismutation of superoxide (O^2−^) [12]; therefore, it acts as an antioxidant agent. Additionally, an in vivo study demonstrated that zinc can protect against phosphate-induced arterial calcification by inducing the production of a zinc-finger protein and tumor-necrosis-factor-α-induced protein 3 (TNFAIP3), suppressing activation of nuclear factor kappa-light-chain-enhancer of activated B (NF-κB) [13]. Given the various important roles of zinc, it is not surprising if mortality in patients undergoing hemodialysis is affected by zinc deficiency at least partly.

So far, information is limited regarding the possible association of serum zinc levels with mortality in patients undergoing dialysis. There are four studies on this topic (Supplemental Table S1). The first was a prospective cohort study of 111 patients including 43 hemodialysis and 68 peritoneal dialysis [14]. The second was a retrospective study that included 61 patients undergoing hemodialysis, among whom 40% were prescribed zinc containing oral nutritional supplement [15]. The other two prospective studies included 142 [16] and 1278 [17] patients with incident hemodialysis, respectively. Thus, no study was conducted in a sufficiently large cohort of prevalent hemodialysis patients. More importantly, these studies reported inconsistent results, presumably due to differences in statistical approach. According to the guideline from the European Society for Clinical Nutrition and Metabolism, it is essential to interpret results of zinc levels in conjunction with changes in serum albumin [18]. Therefore, more studies are needed in patients undergoing maintenance hemodialysis in which serum albumin levels are carefully considered.

The aim of this study was to explore the possible association between serum zinc levels and all-cause mortality in prevalent patients with kidney failure on maintenance hemodialysis.

## 2. Materials and Methods

### 2.1. Study Design and Participants of This Study

The Osaka Dialysis Complication Study (ODCS) was a prospective cohort study in prevalent patients on maintenance hemodialysis. ODCS included 1696 patients on maintenance hemodialysis from 17 dialysis facilities in Osaka Prefecture, Japan [19]. In the ODCS, participants were followed up from 2012 to 2017. Some results from the ODCS have been published [19,20]. The criteria for inclusion and exclusion for patients with hemodialysis were described previously [19]. From the total of 1696 patients who participated in the ODCS, 34 patients were excluded due to missing serum zinc data. Then, the remaining 1662 patients were selected for this analysis.

### 2.2. Ethical Considerations

The ODCS adhered to the Declaration of Helsinki, and the original and revised study protocols were reviewed and approved by the Ethics Committee, Osaka City University Graduate School of Medicine, Osaka, Japan (Approval No. 2219 and Approval No. 2021-029, respectively). All the participants gave written informed consent before participating in the ODCS. The ODCS was registered at UMIN-CTR (UMIN000007470).

### 2.3. Measurement of Serum Zinc

Blood samples were drawn from the arteriovenous fistula just prior to a hemodialysis session at the beginning of the week, three days after the previous hemodialysis session. The serum zinc concentration was measured using a direct colorimetric assay, based on the nitro-PAPS method, using a JCA-BM6050 BioMajesty (JEOL Ltd., Tokyo, Japan) and ESPA ZnII (Nipro Co., Ltd., Osaka, Japan) reagent at a commercial laboratory (SRL Co., Ltd., Tokyo, Japan) [21,22,23]. Coefficients of variation for within-run and between-day precisions were reported to be less than 3.0% [21].

### 2.4. Mortality Data Collection

The outcome of interest was all-cause mortality. In the ODCS, participants were followed up from 2012 to 2017 with a mean ± standard deviation (SD) and median (interquartile range (IQR)) follow-up of 1354 ± 591 and 1825 (870–1826) days, respectively. Among 1662 patients of the present study, 468 died.

### 2.5. Covariates

We considered the following 18 variables as potential confounders: age, sex, body mass index (BMI), dialysis vintage, underlying kidney disease (diabetic kidney disease or not), prior cardiovascular disease (CVD), presence of hypertension, presence of dyslipidemia, smoking habit, serum calcium, phosphate, intact parathyroid hormone (PTH), use of calcimimetics, use of vitamin D receptor activator (VDRA), blood hemoglobin, use of erythropoiesis-stimulating agent (ESA), use of intravenous (IV) iron, C-reactive protein (CRP), and serum albumin.

Hypertension was defined as 140/90 mmHg or higher and/or antihypertensive medication use [24]. Dyslipidemia was defined as non-high-density lipoprotein cholesterol (Non-HDL-C) ≥ 150 mg/dL and/or high-density lipoprotein cholesterol (HDL-C) ≤ 40 mg/dL and/or statin use. These lipid levels derived from the target levels for patients with CKD recommended by the clinical practice guideline of the Japanese Society of Atherosclerosis [25]. In the ODCS, CVD was defined as previously described [19], and the same definition was used for prior CVD, which was included as a covariate in this analysis.

### 2.6. Statistics

The patients for analysis were divided into quartiles of serum zinc levels, and the baseline data were summarized as numbers and percentages for categorical variables or medians and IQR for continuous variables. Categorical variables and continuous variables were compared using χ^2^ test and Kruskal–Wallis test, respectively. In the total patients analyzed, the distribution of serum zinc level was shown using a histogram, and unadjusted correlation of serum zinc with serum albumin was examined by Spearman’s rank correlation test.

The survival difference across the zinc quartiles was examined using Kaplan–Meier analysis with log-rank test. The hazard ratio was calculated using Cox proportional hazards models unadjusted and adjusted for the above-mentioned 18 covariates including serum albumin.

The European Society for Clinical Nutrition and Metabolism guideline states that measuring serum albumin levels is necessary for interpreting zinc levels [18]. In a previous study, Yang et al. categorized their patients with hemodialysis into 4 groups according to their median serum zinc and albumin, because these 2 parameters reflect nutritional status [14]. Then, we categorized our patients into 4 groups according to their median serum levels of zinc and albumin, as was performed in a previous study [14]. The survival difference across these 4 groups was examined using Kaplan–Meier analysis with a log-rank test. The hazard ratios were calculated Cox models with and without adjustment for the 17 covariates excluding serum albumin.

These statistical calculations were conducted using statistical software JMP 14.3.0 (SAS Institute Japan, Tokyo, Japan) and R version 4.3.2 (The R Foundation for Statistical Computing, Vienna, Austria). A *p*-value < 0.05 from a two-sided test was considered statistically significant.

## 3. Results

### 3.1. Characteristics of Study Participants

**Figure 1:**
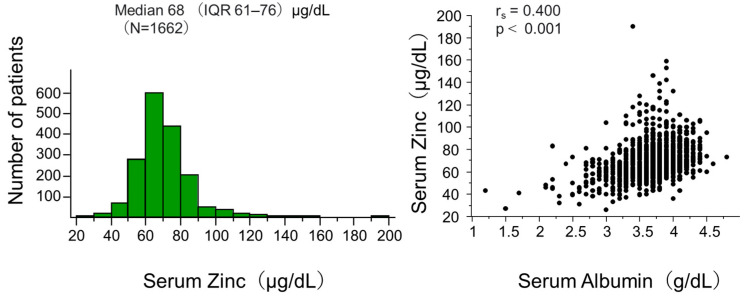
Distribution of zinc and its correlation with albumin at baseline.

Figure 1 (left panel) shows the distribution of serum zinc levels in the total participants of this analysis. The median (IQR) was 68 (61–76) μg/dL, showing an almost normal distribution with a few patients having very high levels.

The left panel of the figure presents the histogram of serum zinc. The right panel shows the correlation between serum zinc and albumin. Abbreviation: IQR interquartile range; N, number of patients; *r_s_*, Spearman’s correlation coefficient; *p*, level of significance.

Table 1 summarizes their baseline characteristics by the quartiles of serum zinc levels. The patients with lower zinc levels had higher age, lower BMI, lower albumin, lower phosphate, lower calcium, and higher CRP, whereas there was no significant difference in hemoglobin among the zinc quartiles.

### 3.2. Serum Zinc Levels and Mortality

**Figure 2:**
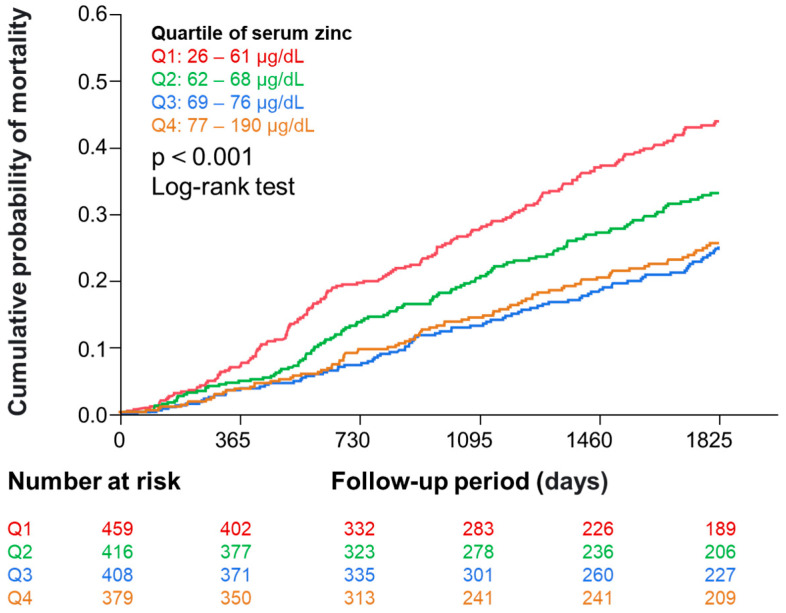
Kaplan–Meier curves showing the association of zinc quartiles with all-cause mortality. Abbreviation: Q, quartile.

During the 5-year observation period, 468 (28%) participants died. Figure 2 shows the Kaplan–Meier analysis indicating that the risk of all-cause mortality was different among serum zinc quartiles.

Table 2 summarizes the association between the quartiles of serum zinc and morality using Cox proportional hazard models. Higher serum zinc levels were found to be associated with a lower risk for mortality, independent of the major demographic factors, factors of mineral and bone disorder (MBD), and factors of renal anemia. However, this association was no longer significant when further adjusted for serum albumin.

### 3.3. Relationship between Zinc with Mortality in Four Groups Categorized by Median Serum Albumin and Zinc Levels

**Figure 3:**
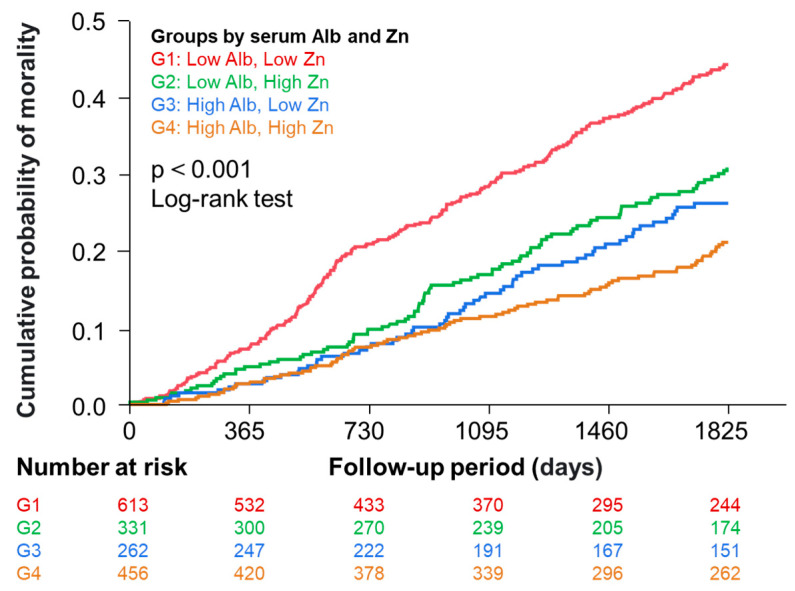
Kaplan–Meier curves showing the association of the four groups divided by median serum zinc and albumin levels with all-cause mortality. Participants were categorized into 4 groups according to their median serum levels of albumin and zinc. Group 1, low (≤3.7 g/dL) albumin and low zinc (≤68 µg/dL); Group 2, low (≤3.7 g/dL) albumin and high zinc (>68 µg/dL); Group 3, high (>3.7 g/dL) albumin and low zinc (≤68 µg/dL); Group 4, high (>3.7 g/dL) albumin and high zinc (>68 µg/dL).

Figure 1 (right panel) shows a significant correlation between serum zinc and albumin levels in the total patients. This could be explained by a well-known fact that approximately 80% of serum zinc is bound to albumin [2], but this could potentially influence the analysis adjusted for albumin. We performed further analyses in which participants were categorized into four groups by median serum zinc and albumin levels. Figure 3 shows Kaplan–Meier curves demonstrating that risk of death was significantly different among the four groups.

### 3.4. Association between Zinc with Mortality in the Low and High Albumin Groups

Table 3 summarizes the association between serum zinc and mortality in the two groups of lower (≤3.7 g/dL) and higher (>3.7 g/dL) serum albumin using Cox proportional hazard models. In the lower serum albumin group, risk of all-cause mortality was higher in the lower serum zinc group (≤68 µg/dL) than in the higher serum zinc group (>68 µg/dL) in analyses with and without adjustment for the 17 covariates. In contrast, the association between zinc and mortality was not significant in the higher serum albumin groups with or without adjustment.

## 4. Discussion

This study examined the possible association of the serum zinc level with all-cause mortality in a prospective cohort of prevalent patients undergoing maintenance hemodialysis. Although the serum zinc level was a significant predictor of mortality in a model adjusted for 17 covariates excluding serum albumin, serum zinc was not a significant predictor of mortality independent of the serum albumin level. To avoid a statistical problem caused by the positive correlation between serum levels of zinc and albumin, we categorized the cohort into four groups according to median serum zinc (68 µg/dL) and albumin (3.7 g/dL) levels. Then, the risk of death was significantly higher in patients with lower zinc than the higher zinc counterparts within the lower serum albumin groups, whereas such a difference was not significant between zinc levels within the higher serum albumin groups. These results indicate that a lower serum zinc level was a significant factor associated with a higher risk of mortality in patients on maintenance hemodialysis, particularly in those with low serum albumin levels.

Since approximately 60–80% of circulating zinc is bound to albumin in serum [2,26,27], serum zinc and albumin concentrations positively correlate with each other as confirmed in this study. Serum albumin is an established nutritional marker that predicts mortality [28,29]. Therefore, it is difficult to distinguish whether the higher risk of mortality is attributable to lower serum zinc itself or lower serum albumin. The guideline from the European Society for Clinical Nutrition and Metabolism also stated that serum albumin levels would be considered in the interpretation of zinc levels [18]. However, the guideline does not specifically mention how to consider serum albumin levels. Four previous studies report the association between serum zinc and all-cause mortality in patients with kidney failure treated with hemodialysis by different statistical approaches as summarized in Supplemental Table S1. The sample sizes of the three studies by Yang et al. [14], Knehtl et al. [15], and Toida et al. [16] were relatively small, and the numbers of deaths ranged between 11 and 15, which did not allow sufficient statistical adjustment and solid conclusion. The study by Tonelli et al. [17] in incident hemodialysis patients was the largest among the four previous studies, and multivariable-adjusted analysis was conducted using a forward stepwise approach by which covariates were selected from many potential confounders including serum albumin. However, by this approach, they showed that lower serum zinc was not a significant factor predicting all-cause mortality. The same was true in our study. When serum albumin was included as a covariate in the multivariable-adjusted Cox model, the association between serum zinc and mortality was no longer significant.

In the subsequent analysis, we handled serum albumin not as a confounder but as a stratifying or grouping factor. We stratified the cohort into two levels by median serum albumin (3.7 g/dL), and the higher and lower serum zinc levels were defined by the median serum zinc level (68 µg/dL). The four groups showed significant differences in mortality risk, and the difference between the higher and lower serum zinc levels remained significant even when adjusted for the 17 covariates excluding serum albumin in the lower serum albumin groups but not in the higher serum albumin groups. These results support the notion that a lower serum zinc concentration is a significant factor associated with a higher risk of mortality in prevalent patients on maintenance hemodialysis, particularly in those with lower serum albumin levels.

There are several possible mechanisms for the observed difference in mortality risk between the higher and lower serum zinc levels based on its involvement in various biological processes as a cofactor of more than 300 enzymes that regulate metabolism [7], blood pressure control [9,10], immune function [11], anti-oxidative mechanisms [12], and vascular calcification [13]. A meta-analysis of 15 randomized controlled trials of zinc supplementation for patients undergoing maintenance hemodialysis showed increases in serum zinc levels and dietary protein intake and improved inflammatory markers including CRP, superoxide dismutase, and malondialdehyde [27]. Additionally, two randomized controlled trials demonstrated a significant increase in serum albumin levels with zinc supplementation at a daily elemental zinc dose of 11 mg for 8 weeks [29] and 100 mg for 60 days [30]. Based on the results of the present study, the association between serum zinc and mortality was significant in patients undergoing hemodialysis with lower serum albumin but not those with higher serum albumin. Therefore, zinc supplementation may be more beneficial in patients undergoing maintenance hemodialysis with hypoalbuminemia and zinc deficiency, although further studies are needed. The European Society for Clinical Nutrition and Metabolism guideline recommended that 0.5–1 mg/kg per day of elemental zinc can be given orally for 3–4 months in patients with acquired zinc deficiency [18]. Further interventional studies are needed to determine whether such zinc supplementation can improve all-cause mortality in patients on hemodialysis.

This study has several limitations. First, because this study included only patients undergoing maintenance hemodialysis in Japan, it is needed to confirm that the results of this study are applicable to other populations. Second, because the results of this study were based on a single measurement of serum zinc, the association between zinc and mortality may be underestimated or overestimated. Third, although blood sampling for the measurement of zinc is recommended in the morning [31], it was not necessarily performed in morning fasting conditions in the ODCS. Fourth, the zinc measurement method used in the present study was the colorimetric assay with the ESPA ZnII reagent, although the European Society for Clinical Nutrition and Metabolism guidelines state that total zinc should preferably be measured using ICP-MS or atomic absorption spectroscopy [18]. However, the serum zinc levels measured by this colorimetric assay were found to be comparable to those obtained using the traditional atomic absorption method [r = 0.971 (*n* = 98)] [32]. Fifth, this study did not include data regarding medications containing zinc (such as polaprezinc and zinc acetate hydrate), dietary intake of zinc, or zinc supplements; therefore, serum zinc levels were not adjusted for these variables. Zinc supplementation increases serum zinc levels in patients on hemodialysis [27]. A prospective cohort study involving 528 patients with hemodialysis demonstrated a 4.1-fold increase in all-cause mortality risk in those with a dietary zinc intake less than 8 mg for women and 12 mg for men [33]. Therefore, the inclusion of these variables may have affected the results. Nevertheless, information regarding these variables has been lacking in the previous studies of this topic [14,15,16,17], including ours. Information on oral nutritional supplements was provided in only one previous study [15]. Further observational studies that include these significant variables are necessary to examine the association between serum zinc levels and all-cause mortality. Sixth, because of the observational nature of this study, the observed associations do not necessarily indicate causality. Nevertheless, the relatively large sample size of this study is one of its strengths.

## 5. Conclusions

In conclusion, this prospective cohort study revealed that an association between a lower serum zinc concentration and a higher risk of mortality in prevalent patients on maintenance hemodialysis, particularly in those with lower serum albumin levels. Further studies are warranted to determine whether zinc intervention can improve mortality in prevalent patients on maintenance hemodialysis, especially those with lower serum albumin levels.

## Figures and Tables

**Table 1:** Baseline characteristics of participants by quartile of serum zinc.

Variable	Unit	Overall (*n* = 1662)	Serum Zinc Quartile				*p* Values across Quartiles
			Q1 (*n* =459)	Q2 (*n* =416)	Q3 (*n* = 408)	Q4 (*n* =379)	
Zinc	μg/dL	68 (61, 76)	57 (52, 59)	65 (63, 67)	72 (70, 74)	83 (80, 90)	<0.001
Age	year	68 (60, 75)	71 (64, 81)	69 (61, 75)	65 (59, 73)	64 (55, 71)	<0.001
Male sex	N (%)	1048 (63.1)	295 (65.4)	262 (37.0)	247 (60.5)	244 (64.4)	0.640
Body mass index	kg/m^2^	21.1 (19, 23.6)	20.6 (18.7, 22.9)	21.3 (18.9, 23.7)	21.3 (19.3, 23.6)	21.5 (19.84, 21.2)	<0.001
Dialysis vintage	month	64 (29, 133)	54 (24, 118)	73 (29, 141)	66 (32, 132)	68 (29, 138)	0.046
Hypertension	N (%)	1498 (90.3)	405 (88.2)	375 (90.3)	374 (91.9)	344 (91.0)	0.305
Dyslipidemia	N (%)	1029 (61.9)	265 (57.7)	272 (65.4)	254 (62.3)	238 (62.8)	0.129
Diabetic kidney disease	N (%)	676 (40.7)	170 (37.0)	162 (38.4)	182 (44.6)	162 (42.7)	0.097
Prior CVD	N (%)	634 (38.1)	190 (41.4)	143 (34.8)	149 (36.5)	152 (40.1)	0.131
Smoker	N (%)	254 (15.3)	59 (12.9)	60 (14.4)	60 (14.7)	75 (19.8)	0.149
Hemoglobin	g/dL	10.6 (10.0, 11.3)	10.6 (9.8, 11.2)	10.7 (10.0, 11.2)	10.6 (10.0, 11.4)	10.6 (10.0, 11.3)	0.222
Use of IV iron	N (%)	443 (26.7)	120 (26.1)	111 (26.7)	112 (27.5)	100 (26.4)	0.976
Use of ESA	N (%)	1369 (82.4)	394 (85.8)	320 (76.9)	343 (95.1)	312 (82.3)	0.004
Calcium	mg/dL	8.9 (8.5, 9.4)	8.8 (8.3, 9.2)	8.9 (8.4, 9.5)	9.0 (8.6, 9.5)	9.0 (8.6, 9.5)	<0.001
Phosphate	mg/dL	5.1 (4.3, 5.9)	4.8 (4.0, 5.8)	5.0 (4.3, 5.7)	5.1 (4.3 5.8)	5.5 (4.5, 6.3)	<0.001
intact PTH	pg/mL	115 (60, 187)	115 (56, 182)	122 (64, 195)	118 (72, 202)	107 (54, 178)	0.066
Use of VDRAs	N (%)	1113 (68.2)	307 (66.9)	272 (65.4)	292 (71.6)	262 (69.1)	0.244
Use of Calcimimetics	N (%)	334 (20.0)	90 (19.6)	99 (23.8)	70 (17.2)	75 (19.8)	0.120
C-reactive protein	mg/dL	0.1 (0.05, 0.32)	0.16 (0.07, 0.64)	0.10 (0.06, 0.31)	0.10 (0.05, 0.24)	0.10 (0.05, 0.21)	<0.001
Albumin	g/dL	3.7 (3.5, 3.9)	3.5 (3.3, 3.7)	3.7 (3.3, 3.7)	3.8 (3.6, 4.0)	3.8 (3.6, 4.0)	<0.001

Data are expressed as numbers, percentages, or medians (interquartile ranges). The range of serum zinc levels for each quartile was 26–61, 62–68, 69–76, and 77–190 μg/dL, respectively. *p*-values were from Kruskal–Wallis test or χ^2^ test. Abbreviations: CVD, cardiovascular disease; ESA, erythropoiesis stimulating agent; IV, intravenous; PTH, parathyroid hormone; Q; quartile; VDRA, vitamin D receptor activator.

**Table 2:** Associations of quartile of serum zinc with all-cause mortality by Cox models.

Variable	Unadjusted			Adjusted Model 1			Adjusted Model 2		
	Hazard Ratio	95% CI	*p*	Hazard Ratio	95% CI	*p*	Hazard Ratio	95% CI	*p*
Zinc	0.77	0.71–0.84	<0.001	0.88	0.81–0.96	0.006	0.95	0.87–1.04	0.300
Age	1.07	1.06–1.08	<0.001	1.07	1.05–1.08	<0.001	1.06	1.05–1.07	<0.001
Male sex	1.13	0.94–1.37	0.197	1.15	0.94–1.42	0.158	1.20	0.98–1.47	0.085
Body mass index	0.91	0.88–0.93	<0.001	0.93	0.90–0.96	<0.001	0.94	0.91–0.97	<0.001
Dialysis vintage	0.99	1.00–1.00	0.183	1.00	1.00–1.00	0.071	1.00	1.00–1.00	0.164
Hypertension	0.82	0.61–1.09	0.171	0.66	0.48–0.90	0.008	0.70	0.51–0.95	0.021
Dyslipidemia	1.08	0.90–1.30	0.421	1.00	0.83–1.22	0.957	0.98	0.80–1.19	0.815
Diabetic kidney disease	1.47	1.22–1.76	<0.001	1.65	1.35–2.02	<0.001	1.68	1.37–2.05	<0.001
Prior CVD	2.29	1.91–2.75	<0.001	1.87	1.55–2.27	<0.001	1.83	1.51–2.22	<0.001
Smoker	0.76	0.57–1.00	0.051	1.16	0.87–1.56	0.317	1.19	0.89–1.60	0.236
Hemoglobin	0.91	0.84–0.98	0.019	1.00	0.92–1.09	0.947	1.06	0.97–1.15	0.215
Use of IV iron	0.94	0.76–1.16	0.565	0.93	0.75–1.15	0.509	0.94	0.76–1.17	0.604
Use of ESA	1.08	0.85–1.38	0.525	1.11	0.86–1.43	0.433	1.09	0.84–1.41	0.520
Calcium	0.78	0.69–0.88	<0.001	0.93	0.81–1.06	0.275	1.05	0.91–1.21	0.491
Phosphate	0.84	0.78–0.91	<0.001	0.97	0.90–1.05	0.470	1.00	0.92–1.08	0.938
intact PTH	1.00	1.00–1.00	0.943	1.00	1.00–1.00	0.999	1.00	1.00–1.00	0.999
Use of VDRAs	0.81	0.67–0.98	0.027	1.00	0.82–1.22	0.978	1.03	0.84–1.26	0.794
Use of Calcimimetics	0.63	0.49–0.82	<0.001	0.88	0.66–1.17	0.387	0.95	0.71–1.26	0.721
C-reactive protein	1.11	1.07–1.15	<0.001	1.10	1.04–1.15	<0.001	1.06	1.01–1.12	0.031
Albumin	0.26	0.21–0.32	<0.001	—	—	—	0.42	0.31–0.56	<0.001

The unadjusted model indicates HR (95% CI) for each variable that was not adjusted for other variables. Adjusted Model 1 was adjusted for age, sex, BMI, DKD or not, duration of HD, prior CVD, hypertension, dyslipidemia, smoking, CRP, calcium, phosphate, intact PTH, use of VDRA use of calcimimetics, Hb, use of ESA, and use of IV iron. In Adjusted Model 2, adjustment was carried out for the variables included in Model 1 + Albumin. Abbreviations: BMI, body mass index; CI, confidence interval; CRP, C-reactive protein; CVD, cardiovascular disease; DKD, diabetic kidney disease; ESA, erythropoiesis stimulating agent; HD, hemodialysis; IV, intravenous; PTH, parathyroid hormone; VDRA, vitamin D receptor activator.

**Table 3:** Associations of serum zinc and albumin with all-cause mortality by Cox models.

Patient Groups		N	Unadjusted			Adjusted		
Serum Albumin (g/dL)	Serum Zinc (μg/dL)		HR	95%(CI)	*p*	HR	95%(CI)	*p*
≤3.7	≤68	613	1.64	1.29–2.10	<0.001	1.31	1.02–1.69	0.036
	>68	331	1.00	Ref.	—	1.00	Ref.	—
>3.7	≤68	262	1.27	0.91–1.77	0.157	1.15	0.82–1.61	0.429
	>68	456	1.00	Ref.	—	1.00	Ref.	—

Adjustment was carried out for age, sex, BMI, DKD or not, duration of HD, prior CVD, hypertension, dyslipidemia, smoking, CRP, calcium, phosphate, intact PTH, use of VDRA, use of calcimimetics, Hb, use of ESA, and use of IV iron. Abbreviations: BMI, body mass index; CI, confidence interval; CRP, C-reactive protein; CVD, cardiovascular disease; DKD, diabetic kidney disease; ESA, erythropoiesis stimulating agent; HD, hemodialysis; IV, intravenous; PTH, parathyroid hormone; VDRA, vitamin D receptor activator.

## Data Availability

The dataset that supports the findings of this study cannot be shared publicly due to ethical restriction for the protection of personal and sensitive information of individuals that participated in the study. The data will be shared on reasonable request to the corresponding author after permission by the ethics committee at our institution is given.

## Supplementary Materials

The following supporting information can be downloaded at https://www.mdpi.com/article/10.3390/nu16193270/s1: Table S1: Studies regarding the association between serum zinc and mortality in patients with dialysis.
