# Supplementary material for: Association between Serum Zinc and All-Cause Mortality in Patients Undergoing Maintenance Hemodialysis: The Osaka Dialysis Complication Study (ODCS)
Source: Nutrients. 2024 Sep 27;16(19):3270. doi: 10.3390/nu16193270 (PMC11478467; doi:10.3390/nu16193270)
Supplement: Supplementary file 1 [file nutrients-16-03270-s001.zip › nutrients-3230114-supplementary.pdf]

Supplemental Table S1. Studies regarding the association between serum zinc and mortality in patients with dialysis.

**Authors**	**Population (N)**	**Number of deaths in follow-up period**	**Statistical approach**	**Conclusion**
Yang CY, et al *Clin Nutr* **2012**, *31*, 630-636 [14]	Prevalent patients on maintenance dialysis (N=111) Hemodialysis (N=43) and peritoneal dialysis (N=68)	14 deaths in 2 years	Backward stepwise Cox analysis	Old age, hypoalbuminemia, and zinc deficiency were independent predictors of mortality.
Knehtl M, et al. *BMC Nephrol* **2022**, *23*, 355 [15]	Prevalent patients on maintenance hemodialysis (N=61)	11 deaths in 2.8 years	Kaplan-Meier method, Univariate Cox model, and multivariable Cox model	No significant association of serum zinc with mortality.
Toida T, et al. *Nutrients* **2020**, *12*, 3187 [16]	Incident hemodialysis (N=142)	15 deaths in 2.5 years	Multivariable-adjusted Cox model including 16 covariates including serum albumin. Serum zinc was handled as three groups (< 45, 45-59, and ≥ 60 μg/dL).	The association between serum zinc levels and all-cause mortality was not clear after adjustments for potential confounders.
Tonelli M, et al. *Clin J Am Soc Nephrol* **2018**, *13*, 907-915 [17]	Incident hemodialysis (N=1278)	260 deaths in 2 years	Forward stepwise logistic model in which serum albumin was included as a candidate variable and serum zinc was handles as an ordinal equidistant decile bins.	Lower level of zinc was not associated with higher risk of death.
Nakatani S, et al [This study]	Prevalent patients on maintenance hemodialysis (N=1662)	468 deaths in 5 years	(1) Multivariable-adjusted Cox model in which serum albumin was adjusted as a confounder and serum zinc was handled as a continuous variable.	(1) Serum zinc was not a significant predictor of mortality independent of serum albumin.

			(2) Multivariable-adjusted Cox model in which both serum albumin and zinc levels were handled as grouping factors.	(2) A lower serum zinc level (≤ 68 μg/dL) was a significant factor predicting a higher risk of mortality in those with lower serum albumin (≤ 3.7 g/dL)
